# A Finite Element Model of Cerebral Vascular Injury for Predicting Microbleeds Location

**DOI:** 10.3389/fbioe.2022.860112

**Published:** 2022-04-20

**Authors:** Harry Duckworth, Adriana Azor, Nikolaus Wischmann, Karl A. Zimmerman, Ilaria Tanini, David J. Sharp, Mazdak Ghajari

**Affiliations:** ^1^ HEAD Lab, Dyson School of Design Engineering, Imperial College London, London, United Kingdom; ^2^ The Computational, Cognitive and Clinical Neuroimaging Laboratory, Imperial College London, London, United Kingdom; ^3^ Industrial Engineering Department, University of Florence, Florence, Italy; ^4^ Care Research and Technology Centre, Dementia Research Institute, London, United Kingdom

**Keywords:** microbleeds, cerebral vasculature injury, traumatic brain injury, finite element model, multibody simulation, microhaemorrhages, cerebrovasculature

## Abstract

Finite Element (FE) models of brain mechanics have improved our understanding of the brain response to rapid mechanical loads that produce traumatic brain injuries. However, these models have rarely incorporated vasculature, which limits their ability to predict the response of vessels to head impacts. To address this shortcoming, here we used high-resolution MRI scans to map the venous system anatomy at a submillimetre resolution. We then used this map to develop an FE model of veins and incorporated it in an anatomically detailed FE model of the brain. The model prediction of brain displacement at different locations was compared to controlled experiments on post-mortem human subject heads, yielding over 3,100 displacement curve comparisons, which showed fair to excellent correlation between them. We then used the model to predict the distribution of axial strains and strain rates in the veins of a rugby player who had small blood deposits in his white matter, known as microbleeds, after sustaining a head collision. We hypothesised that the distribution of axial strain and strain rate in veins can predict the pattern of microbleeds. We reconstructed the head collision using video footage and multi-body dynamics modelling and used the predicted head accelerations to load the FE model of vascular injury. The model predicted large axial strains in veins where microbleeds were detected. A region of interest analysis using white matter tracts showed that the tract group with microbleeds had 95th percentile peak axial strain and strain rate of 0.197 and 64.9 s^−1^ respectively, which were significantly larger than those of the group of tracts without microbleeds (0.163 and 57.0 s^−1^). This study does not derive a threshold for the onset of microbleeds as it investigated a single case, but it provides evidence for a link between strain and strain rate applied to veins during head impacts and structural damage and allows for future work to generate threshold values. Moreover, our results suggest that the FE model has the potential to be used to predict intracranial vascular injuries after TBI, providing a more objective tool for TBI assessment and improving protection against it.

## 1 Introduction

Finite Element (FE) models of the human head have been developed to predict the mechanical response of brain tissue to head loading conditions that produce traumatic brain injury (TBI). Several studies have used these models to predict mechanical strain or strain rate in the brain tissue under head loadings in road traffic collisions, sporting incidents, falls and blast (Zhang et al., 2004; Doorly and Gilchrist, 2006; Kleiven, 2006; Bass et al., 2012; Panzer et al., 2012; Hansen et al., 2013; Ji et al., 2014; Ghajari et al., 2017; Yu et al., 2020). Although FE models can predict the distribution of strain across the brain, only a small number of studies have compared their predictions with the distribution of pathology in humans (Raul et al., 2006; Kleiven, 2007; Ghajari et al., 2017). A recent example is our work where we used an anatomically detailed FE model of the human brain to predict patterns of strain distribution in the brain in three real-life head impacts and showed that large strains were concentrated in the sulci where the pathology of the neurodegenerative disease, chronic traumatic encephalopathy, has been seen (Ghajari et al., 2017). In addition, an analysis of the diffusion-weighted imaging data of a large cohort of single TBI survivors showed white matter abnormalities in the sulci (Ghajari et al., 2017). This FE model, however, lacks a description of vascular anatomy, which limits its ability to predict mechanical response of vessels to loading and explain potential links between impact loading and vascular injuries.

Some computational studies have incorporated superficial and deep vessels in FE models of the human head (Zhang et al., 2002; Ho and Kleiven, 2007; Zhao and Ji, 2020; Subramaniam et al., 2021). The most common inclusion of vasculature has been bridging veins to allow for the study of subdural hematoma, which frequently occurs when the bridging veins rupture (Huang et al., 1999; Kleiven, 2005; Viano et al., 2005; Mao et al., 2013; Cui et al., 2017; Knowles et al., 2017). A few studies used FE modelling to explore the effects of the inclusion of cerebral vasculature on the dynamic response of the brain tissue (Omori et al., 2000; Ho and Kleiven, 2007; Zhao and Ji, 2020). A recent work used a model of vasculature to predict the level of strains in vessels during sporting and road traffic collisions, but the study lacked a description of likely vascular pathologies in the patients (Zhao and Ji, 2021). There are currently no studies that have used an FE model containing a description of vessels to predict the location of cerebral vascular injuries in human. A recent study developed a multi-scale model of vascular injury mechanics in rats and showed that axial strain in vessels predict the areas of blood-brain barrier breakdown following controlled cortical impacts (Farajzadeh Khosroshahi et al., 2021). Here we extend this work to the human to test whether FE models of vascular injury mechanics can predict the location of intracerebral vascular injury seen in human neuroimaging data.

We present the creation of a biomechanically accurate finite element model of the human brain, which includes a high-resolution representation of cerebral veins’ anatomy. We use 7T MRI scans to map details of brain anatomy and the venous system and use a robust methodology to create FE meshes of different tissues involved in load transmission to the vessels, including skull, cerebrospinal fluid (CSF) and brain. The model prediction of brain displacement is then compared with data from post-mortem human subjects (PMHS) subjected to controlled rotations about the three anatomical axes of the head. This comparison allows us to test the accuracy of the model in predicting brain tissue displacement, which can lead to large strains in vessels (Farajzadeh Khosroshahi et al., 2021). To test the ability of the model to predict the location of vascular injuries, we simulate a rugby collision where video footage and SWI (susceptibility weighted imaging) images showing the location of venous injuries, known as microbleeds, are available. (Glushakova et al., 2014; Iwamura et al., 2012; Kenney et al., 2016; Tagge et al., 2018) Microbleeds are a marker of traumatic axonal injury, and their early detection can indicate the severity of trauma (Sharp and Ham, 2011; Lawrence et al., 2017). We reconstruct the collision using multibody dynamics modelling and use the head kinematics to load the FE model of vascular injury. The patterns of axial strain and strain rate in veins are presented and compared to the patterns of microbleeds seen in the SWI image of the player.

## 2 Methods

**FIGURE 1:**
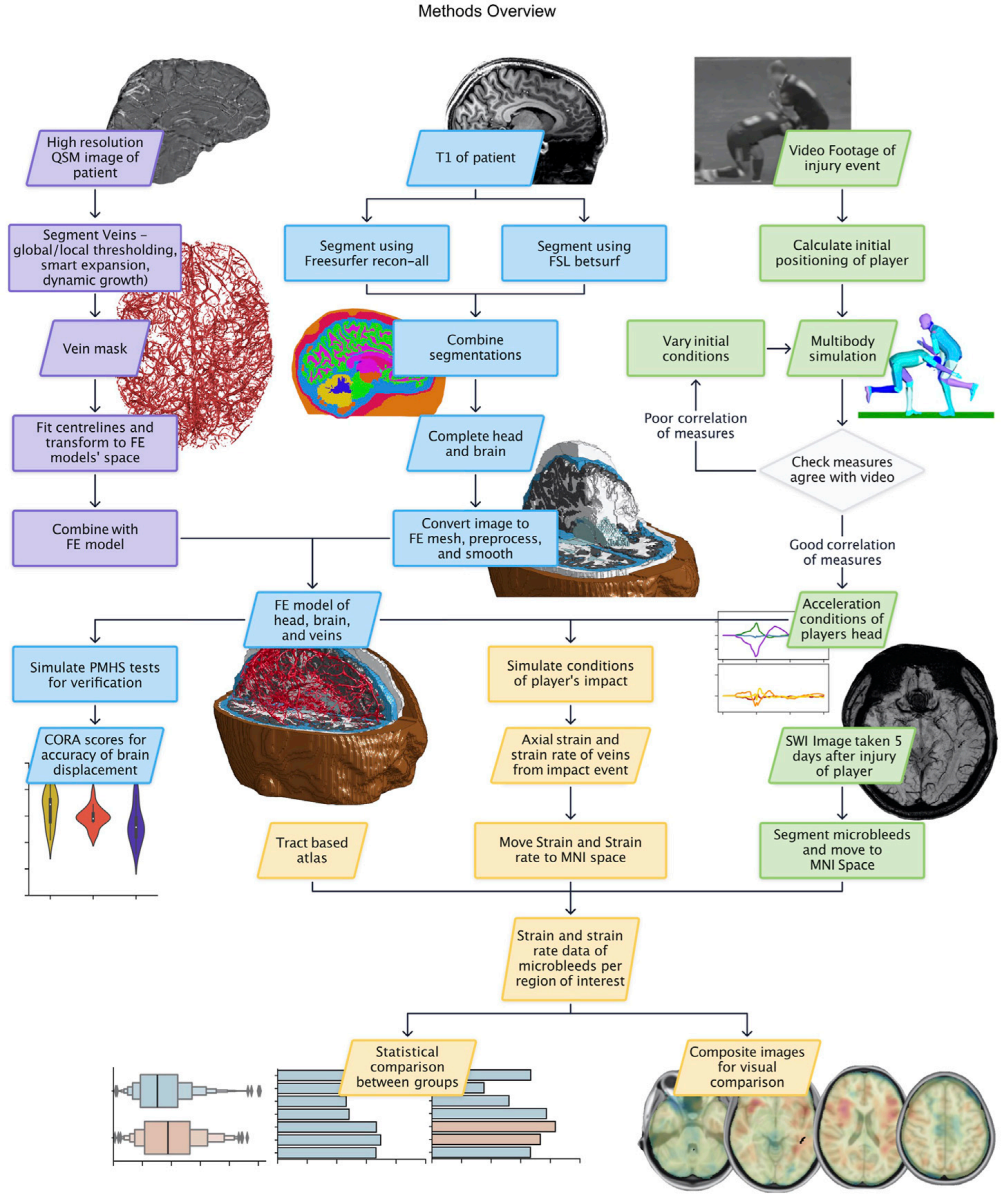
Methods flowchart with data and process boxes coloured according to their theme: vein segmentation in purple, FE model creation in blue, reconstruction in green, and results in yellow.

An overview of the methods is presented in Figure 1.

### 2.1 The Creation of the FE Model of Vascular Injury

#### 2.1.1 High-Field MR Images for FE Model Creation

High-field MRI images of a 34-year-old (at time of scan) healthy Caucasian male subject with no known neurodegenerative or psychiatric disease was acquired using 3T and 7T scanners to create the detailed anatomies of the brain tissue and venous system for FE modelling for this study. The venous system was recreated from ultra-high resolution QSM (quantitative susceptibility mapping) (0.33 mm isotropic) image where a novel prospective motion correction technique was used to improve the resolution of the images for small and large motions (Mattern et al., 2019).

#### 2.1.2 Finite Element Mesh of the Head

The T1 image was resampled to 1.5 mm isotropic voxel size and used to create FE meshes of different tissues of the head using an in-house image-based meshing algorithm upgraded from previous work (Ghajari et al., 2017). First, the cortical and subcortical structures were segmented using freesurfer *recon-all* (v. 7.2.0) (Dale et al., 1999). Then, the scalp and skull were segmented using *betsurf* (fsl v. 6.0.5) (Smith, 2002; Jenkinson et al., 2005) and combined with the freesurfer cortical segmentation to provide masks of brain and CSF. The skull was created by thresholding the T1 image then removing the brain and CSF masks. The CSF mask was expanded twice (using *fslmaths dilM*) to ensure that there is at least one layer of CSF voxels between brain and skull.

An in-house code was used to mesh the anatomy of different tissues using hexahedral elements. To account for the jagged edges at the surface and interfaces between tissues a mesh smoothing algorithm, implemented in another in-house code, was used to smooth the mesh at the scalp surface and interfaces between skull/CSF and CSF/brain. The code controlled for the critical time-step of explicit simulations to avoid a small time-step, which can lead to long simulation times. It also controlled the Jacobian ratio of the elements to avoid small Jacobian ratios, which can lead to computational instabilities. Additionally, the algorithm corrected any lone voxels positioned inside of other parts which if left would lead to errors when smoothing, and it checked for sufficient distances between parts which should not be in contact (white matter against CSF, grey matter against skull, etc.). The FE model was rotated to the Frankfort plane and the centre of gravity was calculated using scaled distances from the occipital condyle described in literature (Yoganandan et al., 2009).

Finally, the dura mater, meshed with quadratic shell elements, was added on the interface between the brain and skull, and the tentorium and falx were added equidistant between the cerebrum/cerebellum and the left/right hemispheres respectively. The shell elements were tied to the solid elements through common nodes.

#### 2.1.3 Finite Element Mesh of the Venous System

The QSM image of the cerebral venous system was segmented using a combination of manual and semi-automatic tools in Mimics (Materialise Mimics Research 20.0) (Figure 1). Thresholding was used to identify macroscale veins (those with diameters larger than approximately 0.5 mm). A dynamic growth algorithm was applied at manually identified vein branches not identified by thresholding, helping to segment smaller veins (<0.5 mm diameter). To ensure continuity of the vein mask and correct edge identification, Mimics’ smart expansion tool was used. Finally, a thresholding brush was applied over sections of the brain where vessels were visible but lacked the contrast to be identified by global thresholding. This allowed for fine details to be achieved due to the fine control over the locations and lengths of veins being selected and prevention of unwanted features being included. The volume of the segmented veins was found to be 2.63% of the total brain volume, which is within the range of values found in literature (Hua et al., 2019; Zhao and Ji, 2020).

**FIGURE 2:**
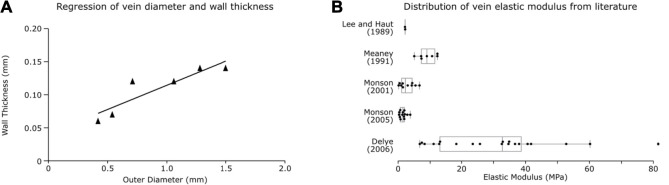
Vein properties. **(A)** Regression plot for vein wall thickness based on outer diameter from Monson (2001). **(B)** Box plots of vein elastic modulus calculated from individual test data found in literature.

Centrelines were fitted to the vein mask with a minimum diameter of 0.33 mm (the voxel size of the QSM) and minimum nodal distance of 1 mm (found to be a reasonable length for simulation efficiency) being specified. The venous system was meshed using beam elements with an annular cross-section. The outer wall diameter of vessels was estimated based on their segmented diameter. A linear regression was found to predict vein’s wall thickness (*h*) from its outer diameter (*d*) based on seven individually measured values from literature (Monson, 2001). We found a strong relationship between the wall thickness and outer diameter ( $R^{2}\;=\;0.8149,\;\;p\;<\;0.001$ , h=0.0732d+0.0411 ), which was used to calculate corresponding wall thickness for the segmented veins based on their outer diameter (Figure 2A).

Finally, we constrained the beam elements of the venous system to the solid elements of the brain matter using the LS-DYNA keyword “*CONTRAINED BEAM IN SOLID” as has been done in previous studies (Ho and Kleiven, 2007; Knowles et al., 2017; Unnikrishnan et al., 2019; Zhao and Ji, 2020; Farajzadeh Khosroshahi et al., 2021). This keyword ties the degrees of freedom of the nodes of beam elements to the displacement vectors of the corresponding points within solid elements.

#### 2.1.4 Material Properties

The venous material properties were based on axial tensile experiments on vivisected and PMHS veins reported in literature. Experiments often report large toe regions of strain changes with little stress, which can be due to relaxation of the vessels pre-testing or innate viscoelastic material properties. To prevent reiteration of material properties leading to over-stiffening due to the embedding of the veins in the viscoelastic brain matter, we did not incorporate the toe region in a material model and instead used an elastic constitutive model for the veins, as has been done in previous studies (Zhang et al., 2002; Ho and Kleiven, 2007). We calculated the elastic modulus from the linear region of the stress-strain curves of individual tests reported in five different studies (Lee and Haut, 1989; Meaney, 1991; Monson, 2001; Monson et al., 2005; Delye et al., 2006) (examples of the region of the curve used is provided in the Supplementary Figure S1 along with the linear response used in this study). This was achieved by dividing the change in stress by change in strain from the linear region of the curve after the toe region (Figure 2B). A one-way ANOVA between the studies showed the significant effect of the study on the elastic modulus of the veins [F (5, 47) = 12.18, *p* < 0.001]. A post hoc Tukey test showed that the Delye 2006 data differed significantly from all other groups (except Löwenheim 1974; where *n* = 1) and that other groups were not significantly different from each other. Therefore, the average of the data reported in other groups was used to define the elastic modulus of the veins in our model (E = 3.63 MPa).

The element stiffness matrix of beam elements requires the definition of the cross section, elastic modulus and Poisson’s ratio. Since the elastic modulus was defined from experiments on vessels, we used this data directly and did not alter them for instance to include potential effects of blood.

Material properties of the grey matter, white matter, brain stem, CSF, skin, skull, and meninges are the same as used in previous work done by this group and can be found in the supplementary material (Supplementary Table S1) (Ghajari et al., 2017).

#### 2.1.5 The Finite Element Model of Vascular Injury

**FIGURE 3:**
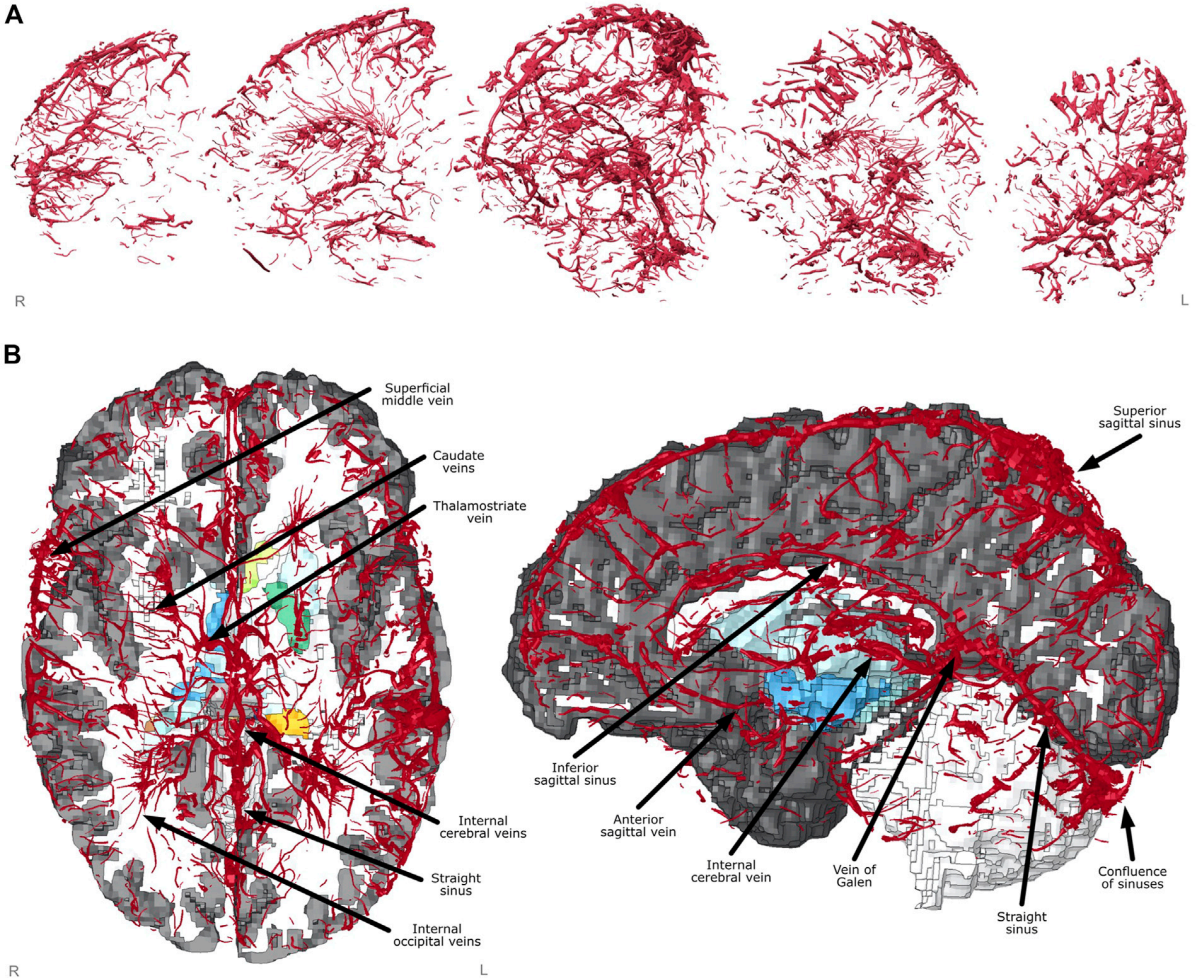
Finite element model creation. **(A)** Vein mesh sagittal cross-sections showing fine detail captured from segmentation and meshing. **(B)** Axial and sagittal views of FE model with sections cut away showing labelled venous system and subcortical structures underneath (coloured).

The FE model of veins included unprecedented details of the anatomy, including many microscale veins in areas perforating through the deep brain such as the caudate veins, the thalamostriate veins and the internal occipital veins, as well as macroscale features such as the superior sagittal sinus, the inferior sagittal sinus, the vein of Galan and the straight sinus (Figures 3A,B). These were identified using the veins in proximity of anatomical locations of subcortical structures, with size and shape cross referenced against a neurosurcical atlas (Felten et al., 2016).

The model contained 1,206,173 elements of which there were 997,019 solid elements, 185,891 shell elements and 23,263 beam elements, and it contained 1,063,631 nodes. The smallest characteristic length of solid, shell and beam elements was 0.190, 0.239, and 0.135 mm respectively and their largest size was 2.225, 1.671 and 4.677 mm. Only 2% of the solid elements had an aspect ratio larger than 3 and 16% had a Jacobian ratio smaller than 0.5, which indicate a good mesh quality. The initial stable time step was 704 nanoseconds, which was dictated by the CSF. A 50 ms simulation on a High-Performance Computer (HPC) cluster with 32 nodes and 24 GB of RAM requested resulted in 4 h of computational wall-time.

### 2.2 Predicting Brain Tissue Displacement in PMHS Heads Subjected to Rotation

Data from controlled PMHS experiments are usually used to validate the predictions of FE models of the human head. However, currently there are no PMHS experiments that have reported intracerebral vascular injuries. Instead, there are several experiments that have measured brain displacement relative to the skull under impact loading (Hardy et al., 2001; Hardy et al., 2007; Feng et al., 2010; Alshareef et al., 2018; Alshareef et al., 2020). Brain tissue displacement can lead to stretching of cerebral vasculature and their damage (Farajzadeh Khosroshahi et al., 2021). Therefore, validating the displacement prediction of the model can build confidence about its ability to predict stretch in vessels.

To validate the model predictions of brain displacement, we used a recent set of experiments where PMHS heads were subjected to controlled rotations about three anatomical axes of the head and brain tissue displacements were measured using 24 receiving crystals implanted in the brain and tracked using the sonomicrometry method (Alshareef et al., 2018; Alshareef et al., 2020). We simulated 46 experiments from five PMHS heads [patient IDs 846, 896, 900, 902, 904 from Alshareef et al. (2020)]. For each PMHS head, the FE model was scaled to match the subject’s head length, width and breadth. The scalp and skin were defined as rigid bodies slaved to the centre of gravity of the head, which was moved to the location described in the experiments. Rotational acceleration pulses measured during the experiments were applied to the centre of gravity. We simulated two rotational velocities, including 20 and 40 rad/s, about the three anatomical axes of the head, and with two pulse durations of 30 and 60 ms. The motion of the brain was recorded at the nodes closest to the respective receiving crystal coordinates. These displacement data were then compared to the reported displacement data using CORA with the settings suggested in (Giordano and Kleiven, 2016). CORA, a widely used method in biomechanics research, ranks the similarity of two curves based on their phase, magnitude and slope. It provides scores from 0 to 1, where 0 indicates no correlation and 1 represents perfect correlation between experiment and simulation (Gehre et al., 2009).

### 2.3 Predicting the Location of Microbleeds After a Rugby Head Collision

To test the ability of the FE model in predicting vascular injury, we simulated a rugby collision in which the tackling player was diagnosed with microbleeds.

#### 2.3.1 Segmenting the Microbleeds

SWI (susceptibility weighted imaging) images acquired 5 days after the injury indicated the presence of localised signal dropouts suggesting the presence of blood deposits, known as microbleeds. The microbleeds were segmented manually from the SWI image using the in-house software ImSeg. The T1 and T2 images were cross referenced to check for abnormalities in the same location to ensure the absence of other pathologies. The SWI was registered to the T1 image of the brain. The T1 image was skull stripped and registered to the MNI template commonly used in neuroimaging (Evans et al., 1993). The microbleeds segmentation was then transformed to the MNI space using the T1 to MNI transformation.

#### 2.3.2 Reconstructing the Collision

In order to reconstruct the head collision, we used a combination of video analysis, multibody dynamics modelling and FE modelling. First, the video footage of the impact was obtained from the public domain. KINOVEA (version beta 0.9.3), a video analysis software, was used to analyse the video footage and determine the initial positions and velocities of the players.

The multibody dynamics software Simcenter Madymo (version 7.8) was then used to reconstruct the collision (Siemens, 2019). An occupant facet model was used to model the players. The models were scaled according to the size and weight of each player by using the GEBOD anthropometrics database, which allowed us to create an accurate representation of both players involved in the impact (Cheng et al., 1996). The head contact characteristics were defined using data from dynamic experiments on PMHS heads (Yoganandan et al., 1995). The coefficient of friction was 0.73 at shoe/ground interface (Clarke and Carré, 2010) and 0.5 between players (Ramalho et al., 2013).

The effects of two uncertain parameters, the initial rotational velocity of the tackler and the distance between the players, on the kinematics of players were explored using Madymo simulations. The distance between different parts of the players to the ground were measured in the simulation and compared with those measured from the video analysis at two time points: head impact time (approx. 10 ms) and head release time (approx. 100 ms). This comparison allowed us to determine the values that led to the best agreement with the video footage, among all explored values. The predicted head accelerations were then used to load the FE model of vascular injury and predict axial strain and strain rate in the veins for 100 ms from 10 ms before initial head contact.

#### 2.3.3 Comparison Between Predicted Strain/Strain Rate in Veins and Microbleeds

The coordinates of beam element nodes representing the veins were recorded every 0.5 ms and were used to calculate strain and strain rate for each timestep using an in-house code. Maximum values were assigned to voxels of an image of the same dimensions as the model’s T1 image which was used to create the FE model. For voxels which had multiple values associated due to the presence of more than one beam element, the maximum value was selected. This approach allowed us to create 3D images containing the maximum values of strain and strain rate across the venous system. The images were then transformed to the MNI251 space using the transformation from the model T1 image. This approach allowed us to compare distributions of maximal strains and strain rates in veins with patterns of microbleeds which was previously transformed to the MNI152 space.

The images containing predicted strain and strain rate of veins elements show a sparse and patchy distribution of values due to the small diameter of vessels and their distance. For improved readability of data and to enable comparison of peak locations, we applied a maximum filtering (*fslmaths -dilF*) then a mean filtering (*fslmaths -dilM*) using a Gaussian kernel of 5 mm to smooth the data.

To aid in the statistical analysis of the strain and strain rate throughout the brain, regions of interest were used from an atlas. Since microbleeds were confined to the white matter, an atlas was created based on the JHU white matter tractography atlas (Griffin et al., 2019) to classify the location of microbleeds (Supplementary Figure S2). Due to the patchy and sparse nature of the raw (unfiltered unsmoothed) strain/strain rate data, the JHU atlas was expanded once (*fslmaths -dilM*) to increase the number of voxels, and therefore vein elements, within a given region of interest. Any region which contained a microbleed was classed as “with microbleed” and other regions were classed as “without microbleed”. The two groups were compared using the Mann Whitney U non-parametric tests to see if there was a significant difference between the groups. The individual regions of interest were also compared together using a Kruskal-Wallis Test then, if required, a post-hoc Dunn test with Bonferroni *p*-value correction to see how many tracts had significantly different values compared to the other tracts. Summary statistics were also calculated for each tract as well as the 95% percentile strain and strain rate to allow for comparison.

## 3 Results

### 3.1 Marginal to Excellent Correlation Between Predicted Brain Displacement and PMHS Data

**FIGURE 4:**
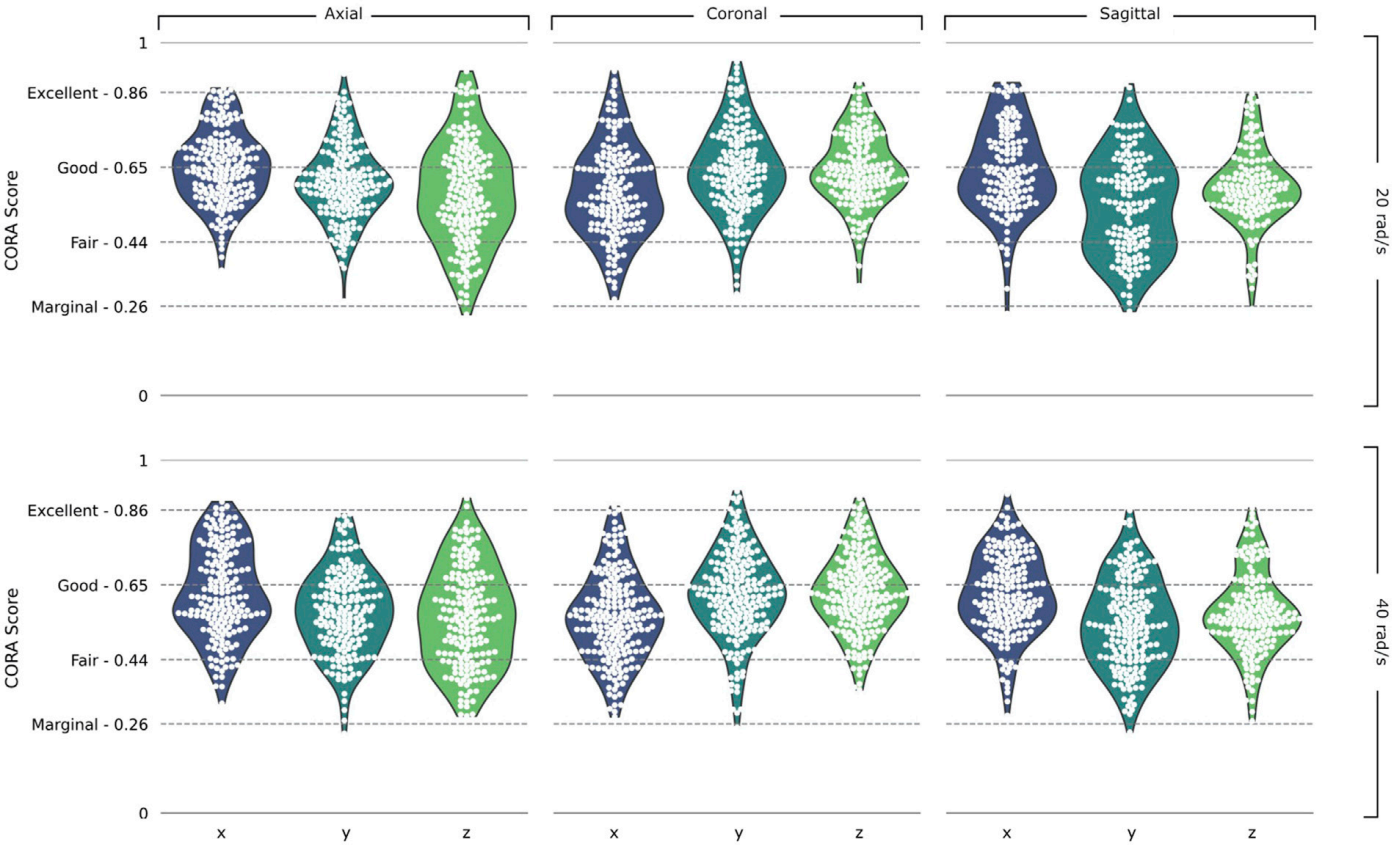
Validation results. Violin plots of CORA scores in the Axial, Coronal, and Sagittal planes and the 20 rad/s and 40 rad/s rotational velocity simulations, split into the x, y, and z components of the head axes. ISO/TR 9790 biofidelity ratings are shown on the vertical axis.

We determined the correlation between displacement time histories predicted by the model and measured in 46 PMHS experiments of five subjects for nearly 24 locations in the brain per test, giving us over 3,100 individual displacement curves each with a corresponding CORA score (Figure 4 and Supplementary Table S1). The scores varied between 0.23 and 0.95 indicating marginal to excellent fidelity according to ISO/TR 9790 biofidelity ratings. The overall average CORA score of all simulations was 0.60, ranking as *fair*. The average CORA scores for the 20 rad/s rotational velocity tests were 0.61, 0.62, 0.59 in the axial, coronal, and sagittal planes respectively. The scores were slightly lower for the 40 rad/s rotational velocity; 0.59, 0.60, and 0.58 in the axial, coronal, and sagittal planes respectively.

### 3.2 Predicted Body Kinematics in the Rugby Collision

**FIGURE 5:**
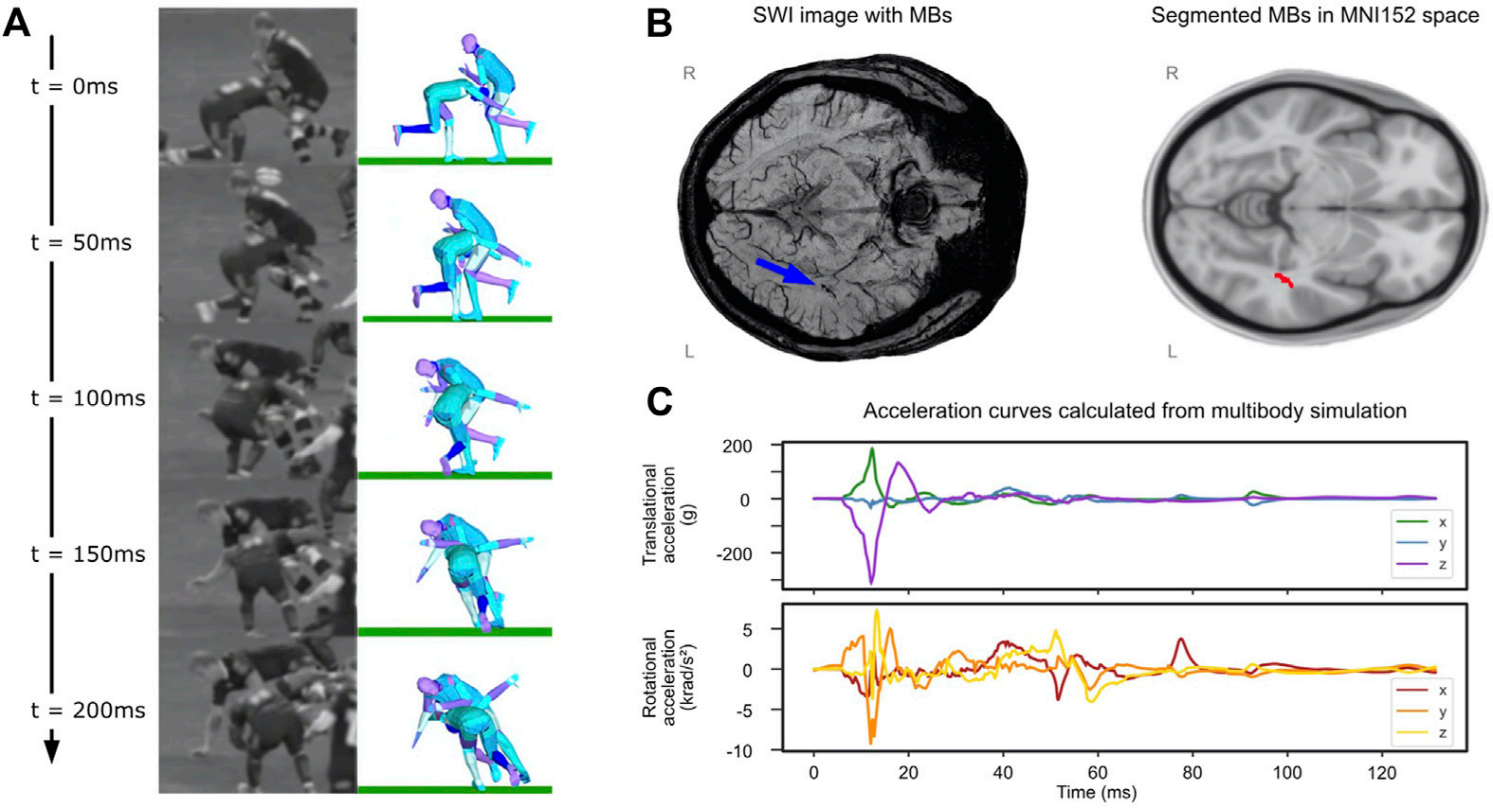
Rugby collision results. **(A)** Comparison of reconstruction to video at 50 ms intervals, patient of interest is tackling player on the left at t = 0. **(B)** (left) SWI image with microbleeds identified by blue arrow and (right) segmented microbleed in MNI5152 space. **(C)** Translational and rotational accelerations of player’s head taken from the multibody simulation.

The video analysis estimated a 7.74 m/s speed along the pitch length for the tackler. The speed of the ball-carrier was estimated as 4.0 m/s along the pitch length and 5.2 m/s along the pitch width. The tackler made the first contact with ball-carrier’s left thigh on the right side of his head and his head was turned to his left (Figure 5A). The analysis of the uncertain parameters showed that the highest Pearson’s correlation coefficient for the comparison between body parts/ground distances measured from the video and simulation was 0.9. Head accelerations for this simulation are shown in Figure 5C. There are large peak translational accelerations in posterior-anterior (x axis) and superior-inferior (negative z axis) directions at around 10 ms. There are also large peak rotational accelerations about the left-right (y) and inferior-superior (z) axes.

### 3.3 Location of Microbleeds in the Tackler

Microbleeds were confirmed by radiologists reports to be located in the left temporal stem (Figure 5B). Where microbleeds were stated as possible, but unconfirmed and not identifiable on SWI during the segmentation, these were not included in subsequent analysis. Microbleeds in the left temporal stem overlapped with the left inferior longitudinal fasciculus (ILF) and left inferior fronto-occipital fasciculus (IFF) white matter tracts.

### 3.4 Large Axial Strain in Veins at the Location of Microbleeds

**FIGURE 6:**
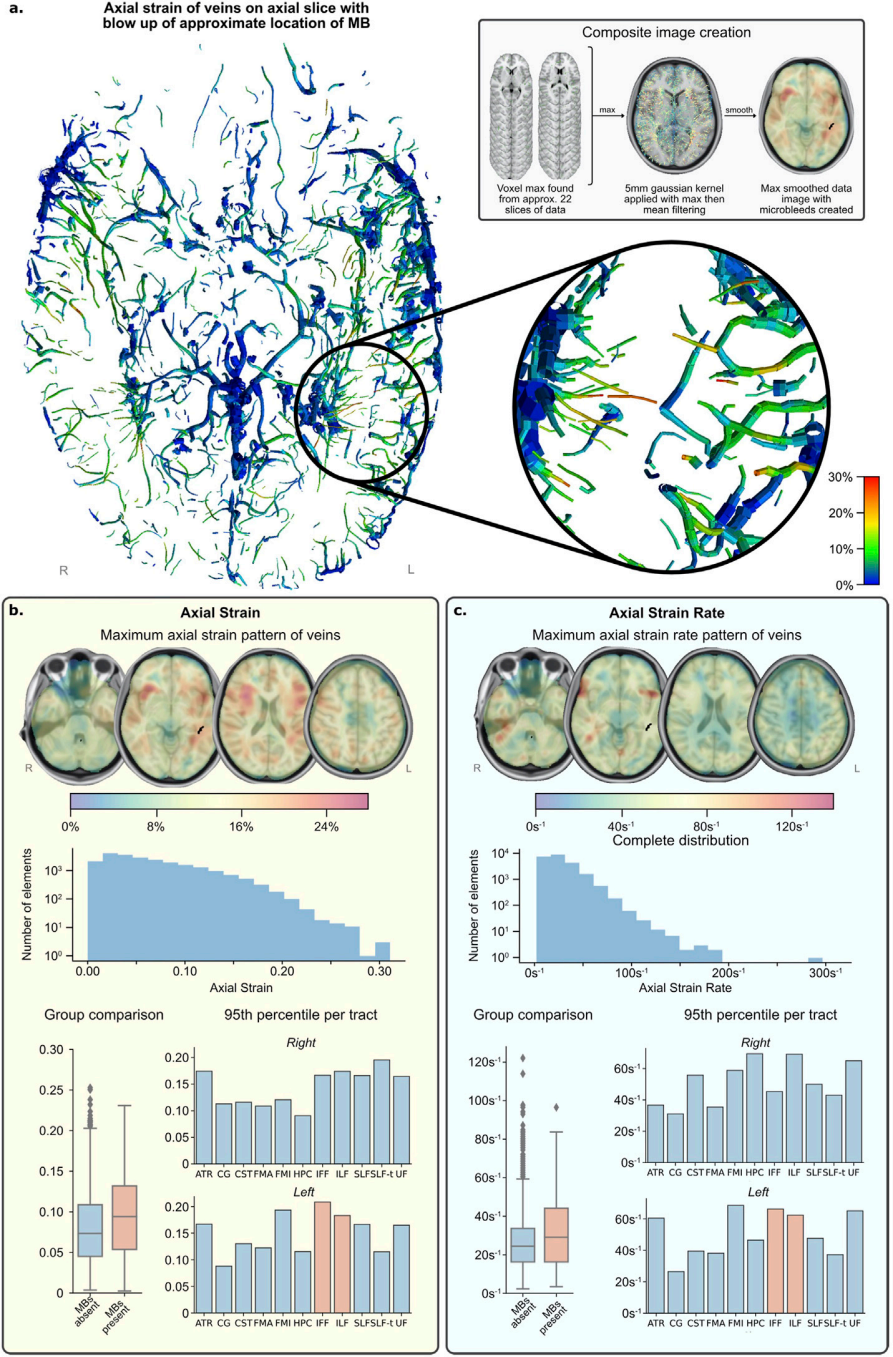
FE simulation results. **(A)** Axial slice of inferior section of veins with maximum strain over time fringe colours. (lower right) Zoomed area of left temporal region shows several elements reaching peak strain levels (0.25–0.30) and is the approximate location of the MBs (upper right) Grey box shows image processing technique used to create composite images in **(B,C)**. **(B)** Vein axial strain results. **(C)** Vein axial strain rate results. NB. Strain and strain rate results **(B,C)**—henceforth called the data—present results in the same format: (top) composite images showing the pattern of data across all time with values calculated from maximum and mean filtering of raw data for visualisation; (middle) histogram of distribution of data from all vein elements in the simulation, not just those present in regions of interest; (bottom left) distribution of grouped regions with MBs compared to those without; (bottom right) 95th percentile of data per tract and per side, regions of interest with MBs present are highlighted in orange. Please see the Supplementary Figure S2 for the definition of the tracts.

The distribution of axial strain of the vein beam elements is shown in Figures 6A,B. Vein elements in the region of the microbleeds have large peak strains in excess of 0.2 (shown by magnified region in Figure 6A). This value is near the higher value of strains predicted in vein elements, which fall between 0 and 0.3, are skewed left and appear to decrease in frequency exponentially (see the axial strain histogram in Figure 6B).

For quantitative comparison, strain data was grouped by tracts. The tracts with microbleeds present had a median strain value of 0.094 and those without had a median strain of 0.073, and the distribution of these groups differed significantly (Mann–Whitney U = 402,191.5, n1 = 345, n2 = 2,732, *p* < 0.001 two-tailed) (Figure 6B; Table 1). A Kruskal-Wallis H-test was completed on the tract-based strain distributions and found groups to be significantly different [*H* (21) = 407, *p* < 0.001], therefore a Post hoc Dunn test was carried out to test for significant difference between tracts. The two tracts which intersected the microbleed, IFF L and ILF L, were found to be significantly different from approximately half and two thirds of the other tracts, respectively, which was also true for fifteen other tracts (Supplementary Table S1). The 95th percentile strain for the two tracts with microbleeds, the IFF L and ILF L, were respectively 0.208 and 0.183, among the highest of all tracts. No other tract reported a higher 95th percentile strain than that seen in the IFF L. However, the FMI L and the SLF-t R reported higher 95th percentile strains than those in the ILF L, the other tract with microbleeds.

**TABLE 1:** Summary statistics of strain per microbleed and non-microbleed grouped tracts.

Strain	Microbleeds	No Microbleeds
*n*	345	2,732
95th Percentile	0.197	0.163
Median	0.094	0.073
Mean	0.096	0.080
Standard deviation	0.053	0.044

### 3.5 Large Axial Strain Rate in Veins at the Location of Microbleeds in the Rugby Collision

The distribution of axial strain rate of the vein beam elements is shown in Figure 6C. High strain rates can be seen in frontal parts of the temporal stems and distributed in no clear pattern in the occipital and temporal lobes. The axial strain rates of vein elements in the region of the microbleeds are more than 60 s^−1^. The strain rate values across the venous system fall between zero and 200 s^−1^ (with a single erroneous value of almost 300 s^−1^). Their distribution is skewed left and appears to decrease in frequency exponentially and steeper than strain.

When grouping tracts by presence of microbleeds, those with microbleeds present had a median strain rate value of 29.1 s^−1^ and those without had a median strain of 24.2 s^−1^ (Table 2). The distribution of these groups differed significantly (Mann–Whitney U = 40,219.5, n1 = 345, n2 = 2,732, *p* < 0.001 two-tailed). A Kruskal-Wallis H-test was completed on the tract-based strain rate distributions and found groups to be statistically significant [*H* (21) = 407.3, *p* < 0.001], therefore a Post hoc Dunn test was carried out to test for significant difference between tracts. The two tracts which intersected the microbleed, the IFF L and the ILF L, were found to be significantly different from 7 to 11 other tracts respectively (out of a total of 20 non-microbleed tracts). Only six other tracts were found to have a similar level of significant differences (>10 significant differences).

**TABLE 2:** Summary statistics of strain per microbleed and non-microbleed grouped tracts.

Strain rate (s^−1^)	Microbleeds	No microbleeds
*n*	345	2,732
95th Percentile	64.9	57.0
Median	29.1	24.4
Mean	32.1	27.2
Standard deviation	18.2	15.4

The 95th percentile strain rate for the two tracts with microbleeds, the IFF L and ILF L, were respectively 66.3 s^−1^ and 62.4 s^−1^, among the highest of all tracts (Supplementary Table S1). There were however three tracts that had 95th percentile strain rates higher than IFF L and five tracts with higher values than ILF L.

## 4 Discussion

This study presents a new finite element model of traumatic brain injury, which incorporates very fine details of the cerebral veins’ anatomy. This model allowed us to predict the distribution of axial strain and strain rate across the venous system, particularly in small veins. We incorporated the venous system in the model to allow us to test whether the model can predict the location of microbleeds, which are small deposits of venous blood seen in SWI scans and their presence can indicate the significance of white matter injury. Simulation of a reconstructed rugby head collision predicted large axial strain in vein elements surrounding the location of microbleeds in player’s white matter, seen in SWI images a few days post-injury. This provides evidence for a link between large axial strains in small cerebral veins and microbleeds, as suggested by previous clinical and pathological studies (Ricciardi et al., 2017; Monson et al., 2018; Griffin et al., 2019). Microbleeds appear rapidly after a TBI event and stay consistent for years after (Graham and Sharp, 2019; Rizk et al., 2020), and their early detection can indicate the severity of trauma (Lawrence et al., 2017; Griffin et al., 2019).

The venous system representation created in this work is the most detailed included in an FE model, allowing for the prediction of small vein injuries. When considering FE models which include more than the bridging veins and connected sinuses, we found three other 3D human head models presented in studies by Ho and Kleiven (2007), Zhao and Ji (2020) and Subramaniam et al. (2021). The Ho and Kleiven model included the largest veins (smallest radii 0.25 mm) which are connected to the sinuses, with a good definition of arteries (smallest radii 0.41 mm). Zhao and Ji’s model included detailed veins and arteries which were limited by the resolution of the source image of 0.60 mm. This model was based on a probabilistic atlas, rather than a specific subject whose MRI images were used to create the FE brain and head as was done in this paper. The Subramaniam et al. model contained veins and arteries with minimum radii of 0.26 and 0.12 mm respectively. Here we have presented an FE brain model with vein radii down to 0.165 mm, which to the authors knowledge, is the highest resolution representation of the venous system used in finite element modelling. The model presented here also uses the same individual for both brain model and venous system, reducing any errors stemming from differences in anatomy between the individual on which the FE brain is created from and the source of the vasculature.

Another novelty of this work is that it shows a link between large strain applied to cerebral veins and their injury. Other studies have investigated bridging vein biomechanics and associated pathology of subdural haemorrhage (Kleiven and Hardy, 2002; Takhounts et al., 2003; Cui et al., 2017; Zhou et al., 2018; Zhou et al., 2019) or investigated the effect of intracerebral vasculature on the mechanical response of the brain (Zhang et al., 2002; Ho and Kleiven, 2007; Zhao and Ji, 2020; Subramaniam et al., 2021). For instance, Ho and Kleiven (2007) found that inclusion of vasculature did not have any significant effect on maximum principal strain of elements representing the brain tissue, but the vasculature resolution was low. Zhao and Ji (2020) recreated these tests with their model, however when they adjusted the material properties, they found significant reductions in maximum principal strain. These works show that both a detailed representation of vasculature and material properties can influence the prediction of strains. However, a comparison between the predictions of vessel deformation and vascular injuries seen in human data was lacking in these previous studies.

When comparing between tracts with and tracts without microbleeds statistically significant differences were seen for both strain and strain rate. 95th percentile strain of the tract group with microbleeds was 0.197, 21% higher than tract group without microbleeds. 95th percentile strain rate of the tract group with microbleeds was 64.5/s, 14% higher than the group without microbleeds. The predicted 95th percentile axial strain values fall within the values reported in current literature. Recent FE modelling of TBI which included intracerebral vasculature reported 95th percentile axial vein strains to be between 0.07–0.20 across five different impacts of varying severities (Zhao and Ji, 2021). Bridging veins, which have been investigated more frequently, report values between 0.17–0.41 maximum axial strain (Zhou et al., 2018), 0.006–018 maximum axial strain (Knowles et al., 2017), and approx. 0.17 maximum principle strain (Subramaniam et al., 2021). To our knowledge there is currently no work which investigates the threshold values which indicate damage for human intracerebral vasculature. A previous work on rats found that intracerebral vessel axial strains of 0.14 were associated with breakdown of the blood brain barrier (Farajzadeh Khosroshahi et al., 2021). In human, physical axial testing of the superficial and bridging veins may give the best indication of thresholds for damage. Axial yield strain of veins can vary between the location and segment used: cortical veins were seen to fail between 0.15–0.73 (Monson, 2001; Monson et al., 2003; Monson et al., 2005), bridging veins at 0.29 ± 0.09 (Monson et al., 2005), and bridging veins with a section of the super sagittal sinus between 0.07–0.18 (Delye et al., 2006; Baeck et al., 2012; Monea et al., 2014). Our 95th percentile values of tracts with MBs fall in the range of cortical vein yield strains, yet some tracts without MBs also are within this range. No yield values exist for the intracerebral vasculature, so it is not possible to draw a direct comparison of like-for-like data. Even so, our results can provide useful information for setting up *ex-vivo* and *in-vivo* experiments to determine strain and strain rate thresholds for damaging veins. In this context, it should be noted that the vast majority of experiments and modelling efforts have focused on measuring axial stress-strain response of vessels. Recent studies however have predicted circumferential strain in vessels as a potential cause of vascular damage, which can be a direction for future studies (Zhao and Ji, 2021).

The accuracy of our model was assessed using 46 post mortem head tests where acceleration curves were provided and target displacements recorded (Alshareef et al., 2020). The overall CORA score from all tests was 0.60, which is ranked *Fair,* however individual target scores ranged from marginal (>0.26) to excellent (>0.86). Results were seen to be consistent between each plane of rotation, rotational velocity and degree of freedom, allowing for trustworthiness of simulation of complex accelerations in each plane and direction. For comparison, a recent study assessed seven brain models using the CORA ranking system and found overall score to be between 0.26 and 0.41 on average (Miller et al., 2017). Another study used the same experiments simulated here to determine a weighted CORA score, based on combining the CORA scores of x, y and z displacement components of the targets (Alshareef et al., 2021). They found highest average weighted CORA scores ranging from 0.36 to 0.63 for three different brain models. Previous works have indicated that both a detailed representation of vasculature and accurate material properties may help in improving the accuracy of FE models prediction (Zhang et al., 2002; Zhao and Ji, 2020; Subramaniam et al., 2021). This may suggest that the inclusion of the venous system may have an influence on the accuracy of the model. In addition, here we use an element size of approx. 1.5 mm for solid and shell elements (before smoothing), which we find to have an acceptable level of resources required. Additionally, we calculate the centre of gravity of the head based on data from literature (Yoganandan et al., 2009), ensuring minimal error in the location where the accelerations are applied. These factors may have contributed to improving the accuracy of the model predictions.

In previous studies a constant wall thickness was applied to veins and arteries irrespective of their outer diameter (Zhao and Ji, 2020; Subramaniam et al., 2021). This would likely lead to unrealistic stiffening of the smallest veins and relaxation of the largest. Here we determined a linear correlation between outer diameter and thickness of veins using the data from Monson (2001). This allowed us to assign different thickness to vessels depending on their diameter. This approach is likely to improve the prediction of axial strain and strain rate that veins undergo during impacts and can be useful for the development of finite element models of the cerebral venous system.

This study has some limitations. The pia-arachnoid complex, plays an important part in transfer of stresses and strains form the skull into the brain, however it is commonly simplified in brain injury models and referred to as the CSF. Having an anatomically accurate distance between the brain and the skull can artificially stiffen the area when under high strains due to the small number of elements present. Methods such as smoothed particle hydrodynamics (Duckworth et al., 2021) and arbitrary Lagrangian–Eulerian (Zhou et al., 2018) have been proposed to model this area more accurately. However, to reduce necessary resources we chose to use the traditional solid element modelling method and accounting for the stiffening problem by artificially expanding the CSF by one voxel, which led to good predictions of brain displacements. Accurate modelling of the CSF layer in FE models of TBI still remains one of the key challenges of this field and more work is required to improve models without compromising the representation of important anatomical features.

We utilise a single value for elastic modulus for the veins, which applies to all elements including the intracerebral veins, bridging veins, and superior sagittal sinus. In literature the material properties of the cortical veins, bridging veins, and superior sagittal sinus have been seen to vary (Monson et al., 2005; Delye et al., 2006; Baeck et al., 2012; Monea et al., 2014). In this work, however, we are primarily focused on the intracerebral veins and their response. The material properties of intracerebral veins are unknown, and we know of no study to investigate this as of yet. Instead, we found material properties from relevant tests which accounted for both cortical and bridging veins, to give the most comprehensive estimation we could achieve. Overall, our value of 3.6 MPa was similar to other models which used a linear elastic material model for vessels (Omori et al., 2000; Ho and Kleiven, 2007; Zoghi-Moghadam and Sadegh, 2009; Mao et al., 2013; Knowles et al., 2017).

We tried to limit the effect of small regions of interest by dilating the original atlas to include more voxels and therefore include more elements within the local region. Despite this, some regions had fewer than 40 elements present: the UF R, SLF-t R, SLF-t L, HPC L, and the FMA R. These tracts have a very high, or very low, sum of significant differences from the Dunn test, and their range of minimum strain and strain rate values is significantly higher than those with greater than 40 elements, possibly indicating a lower quality of this data which may lead to incorrect summary statistics. Excluding these tracts for this simulation leads to an almost negligible, but still apparent, increase of 0.002 in the 95th percentile average of non-MB tracts. Similar changes are seen for the median (0.003 increase) and the mean (0.002 increase). 95th percentile and median strain rate are unaffected by excluding these tracts and mean strain rate increases by 0.01 s^−1^. Although here the effect of tracts with a low count of elements is negligible, under different boundary conditions it may be worth considering their effect as the influence could be more pronounced.

In summary, we have presented a new FE model of venous injury prediction, featuring fine details of the venous system. Large axial strains and strain rates in veins in the location of microbleeds seen in the rugby player suggest a link between the biomechanical loading of veins and the occurrence of vasculature damage. When grouped into tracts with microbleeds and tracts without, a significant difference in distribution can be seen. When considering individual tracts those with microbleeds were consistently seen to have higher strains and strain rates summary statistics when compared to tracts without. Through reconstruction of more TBI cases with microbleeds, we can further prove the efficacy of the model and determine thresholds of strain and strain rate for the prediction of microbleeds, an important TBI biomarker. This FE model has the potential to be used to predict intracranial vascular injuries after TBI, providing a more objective tool for TBI assessment and improving protection against it.

## Data Availability

The original contributions presented in the study are included in the article/Supplementary Material, further inquiries can be directed to the corresponding author.
