# Supplementary material for: A Finite Element Model of Cerebral Vascular Injury for Predicting Microbleeds Location
Source: Front Bioeng Biotechnol. 2022 Apr 20;10:860112. doi: 10.3389/fbioe.2022.860112 (PMC9065595; doi:10.3389/fbioe.2022.860112)
Supplement: Supplementary file 1 [file DataSheet1.docx]

Supplementary Material

Figure S1 shows the elastic modulus calculated for our study and most of the curves used to calculate the elastic modulus. However due to some of the earlier studies not publishing the stress-strain plots these curves are unfortunately not comprehensive. They are beneficial to see how the magnitude of the toe regions varies as well as the differences in slope between certain tests (two of which are shown in red). Direct comparisons between our stress-strain relationship and individual curves from literature should be discouraged however, as these tests are from ex-vivo testing with various methods of acquisition and storage, a reason for our assumption of not modelling the toe region. There are arguments both for and against inclusion of this region; however the limited tests available and lack of a specific study investigating this make it necessary to make these assumptions until better data becomes available.


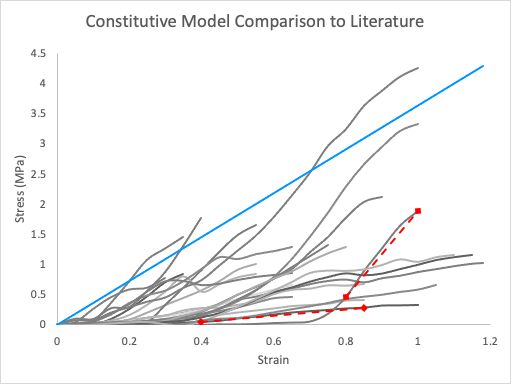


**Supplementary Figure S1 The stress-strain relationship for veins used in this study (blue) and data from literature (grey) from Monson et al. 2003 and Monson et al. 2005 with examples of regions extracted for the elastic modulus estimation (red).**


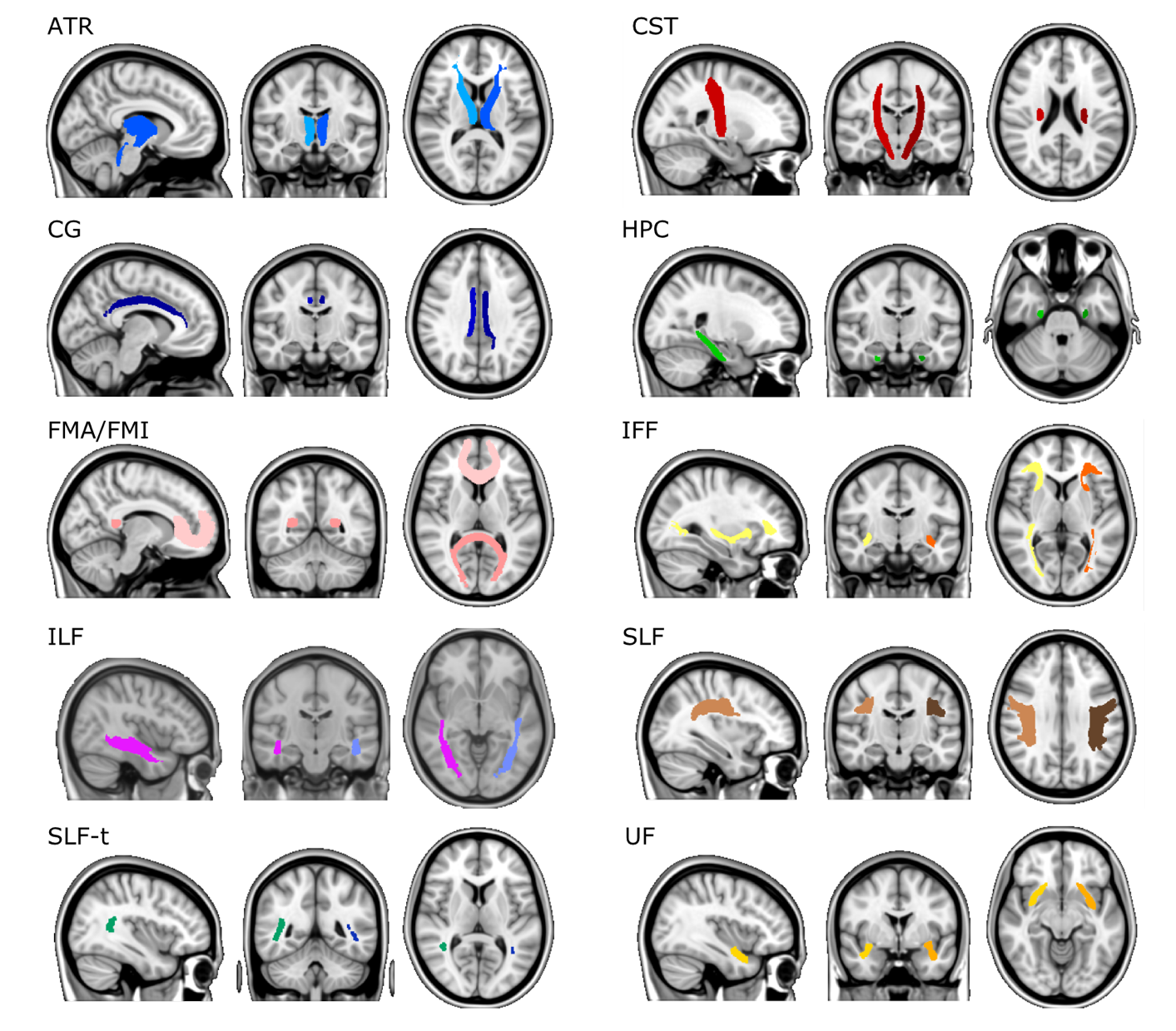


Supplementary Figure S1 Regions of interests, based on JHU white matter tract atlas, used for quantitative comparisons of strain and strain rate. Short and long names in alphabetical order: ATR, Anterior thalamic radiation; CST, Corticospinal tract; CG, Cingulate Gyrus; HPC, Hippocampus; FMA, Forceps major; FMI, Forceps Minor; IFF, Inferior fronto-occipital fasciculus; ILF, Inferior longitudinal fasciculus; SLF, Superior longitudinal fasciculus; SLF-t, Superior longitudinal fasciculus (temporal part); UF, Uncinate fasciculus

Supplementary Table S1 Material properties of brain tissue as used in previous studies by this group.

| Tissue | Density [kg/m3] | Poisson’s Ratio | 𝜇1 [Pa] | 𝛼1 | 𝜇2 [Pa] | 𝛼2 | Bulk modulus [MPa] | 𝜏𝑖 [ms] | 𝐺𝑖 [kPa] |
| --- | --- | --- | --- | --- | --- | --- | --- | --- | --- |
| Brain | 1040 | - | 53.8 | 10.1 | -120.4 | -12.9 | 50 | 𝜏1 =0.001  𝜏2 =0.01  𝜏3 =0.1  𝜏4 =1  𝜏5 =10  𝜏6 =100 | 𝐺1 =320  𝐺2 =78  𝐺3 =6.2  𝐺4 =8.0  𝐺5 =0.1  𝐺6 =3.0 |
| Brain Stem | 1040 | - | 15.8 | 28.1 | -106.8 | -29.5 | 50 |  |  |
| Falx, Dura, Tentorium | 1130 | 0.45 | 25.5 | 2 | - | - | - | 𝜏1 =5  𝜏2 =44  𝜏3 =474 | 𝐺1 =328  𝐺2 =291  𝐺3 =161 |
| Pia Mater | 1130 | 0.45 | 2.6 | 32.9 | - | - | - |  |  |
| CSF and ventricles | 1040 | 0.4998 | 20 | 2 | - | - | - | - | - |

Supplementary Table S2 ISO/TR 9790 ratings for overall CORA scores from each test split by plane of rotation, rotational speed, and peak time, and test ID. Each test has their individual CORA scores per axis direction and the overall score for the test.

| Patient ID | Plane | Velocity | Time | ID | x | y | z | Total |
| --- | --- | --- | --- | --- | --- | --- | --- | --- |
| 846 | Axial | 20rad/s | 30ms | 12844 | 0.653 | 0.589 | 0.533 | 0.592 |
|  |  |  | 60ms | 12843 | 0.693 | 0.638 | 0.549 | 0.627 |
|  |  | 40rad/s | 30ms | 12846 | 0.626 | 0.517 | 0.508 | 0.550 |
|  |  |  | 60ms | 12845 | 0.673 | 0.598 | 0.529 | 0.600 |
|  | Coronal | 20rad/s | 30ms | 12848 | 0.562 | 0.631 | 0.657 | 0.617 |
|  |  |  | 60ms | 12847 | 0.616 | 0.622 | 0.635 | 0.624 |
|  |  | 40rad/s | 30ms | 12850 | 0.587 | 0.616 | 0.586 | 0.596 |
|  |  |  | 60ms | 12849 | 0.609 | 0.640 | 0.615 | 0.621 |
|  | Sagittal | 20rad/s | 30ms | 12852 | 0.656 | 0.476 | 0.602 | 0.578 |
|  |  |  | 60ms | 12851 | 0.631 | 0.447 | 0.586 | 0.555 |
|  |  | 40rad/s | 30ms | 12854 | 0.660 | 0.478 | 0.554 | 0.564 |
|  |  |  | 60ms | 12853 | 0.663 | 0.465 | 0.603 | 0.577 |
| 896 | Axial | 20rad/s | 30ms | 12856 | 0.680 | 0.648 | 0.641 | 0.656 |
|  |  |  | 60ms | 12855 | 0.713 | 0.638 | 0.676 | 0.676 |
|  |  | 40rad/s | 30ms | 12858 | 0.624 | 0.612 | 0.611 | 0.616 |
|  |  |  | 60ms | 12857 | 0.732 | 0.647 | 0.642 | 0.674 |
|  | Coronal | 20rad/s | 30ms | 12860 | 0.551 | 0.694 | 0.671 | 0.639 |
|  |  |  | 60ms | 12859 | 0.619 | 0.695 | 0.674 | 0.662 |
|  |  | 40rad/s | 30ms | 12862 | 0.495 | 0.617 | 0.632 | 0.581 |
|  |  |  | 60ms | 12861 | 0.594 | 0.681 | 0.672 | 0.649 |
|  | Sagittal | 20rad/s | 30ms | 12864 | 0.637 | 0.561 | 0.562 | 0.587 |
|  |  |  | 60ms | 12863 | 0.685 | 0.621 | 0.604 | 0.636 |
|  |  | 40rad/s | 30ms | 12866 | 0.601 | 0.593 | 0.508 | 0.567 |
|  |  |  | 60ms | 12865 | 0.693 | 0.596 | 0.588 | 0.626 |
| 900 | Axial | 20rad/s | 30ms | 12868 | 0.573 | 0.568 | 0.517 | 0.553 |
|  |  |  | 60ms | 12867 | 0.651 | 0.615 | 0.517 | 0.595 |
|  |  | 40rad/s | 30ms | 12870 | 0.578 | 0.542 | 0.502 | 0.541 |
|  |  |  | 60ms | 12869 | 0.631 | 0.595 | 0.533 | 0.586 |
|  | Coronal | 20rad/s | 30ms | 12872 | 0.566 | 0.611 | 0.611 | 0.596 |
|  |  |  | 60ms | 12871 | 0.644 | 0.672 | 0.660 | 0.658 |
|  |  | 40rad/s | 30ms | 12874 | 0.562 | 0.606 | 0.600 | 0.589 |
|  |  |  | 60ms | 12873 | 0.609 | 0.612 | 0.610 | 0.611 |
|  | Sagittal | 20rad/s | 30ms | 12876 | 0.654 | 0.534 | 0.581 | 0.589 |
|  |  | 40rad/s | 30ms | 12878 | 0.595 | 0.530 | 0.553 | 0.559 |
|  |  |  | 60ms | 12877 | 0.583 | 0.520 | 0.564 | 0.556 |
| 902 | Axial | 20rad/s | 30ms | 12880 | 0.637 | 0.590 | 0.600 | 0.609 |
|  |  |  | 60ms | 12879 | 0.631 | 0.589 | 0.572 | 0.597 |
|  |  | 40rad/s | 30ms | 12882 | 0.641 | 0.596 | 0.615 | 0.617 |
|  |  |  | 60ms | 12881 | 0.532 | 0.486 | 0.508 | 0.509 |
|  | Coronal | 20rad/s | 30ms | 12884 | 0.525 | 0.560 | 0.598 | 0.561 |
|  |  | 40rad/s | 30ms | 12886 | 0.531 | 0.623 | 0.622 | 0.592 |
|  |  |  | 60ms | 12885 | 0.582 | 0.593 | 0.654 | 0.610 |
|  | Sagittal | 20rad/s | 60ms | 12887 | 0.604 | 0.576 | 0.618 | 0.599 |
|  |  | 40rad/s | 30ms | 12889 | 0.630 | 0.569 | 0.621 | 0.607 |
|  |  |  | 60ms | 12888 | 0.572 | 0.554 | 0.590 | 0.572 |
| 904 | Coronal | 40rad/s | 30ms | 12910 | 0.496 | 0.618 | 0.658 | 0.590 |
| Total |  |  |  |  | 0.613 | 0.590 | 0.595 | 0.600 |

Supplementary Table S3 Summary statistics of strain across all tracts. Tracts which intercept a microbleed are denoted by *. The post hoc Dunn test with Bonferroni correction is summarised in the final column by sum of tracts with significant difference.

| *Tract* | *n* | *Mean* | *Std* | *Min* | *50%* | *95%* | *Max* | *No. sig dif (**Dunn)* |
| --- | --- | --- | --- | --- | --- | --- | --- | --- |
| *ATR L* | 365 | 0.087 | 0.042 | 0.004 | 0.081 | 0.167 | 0.213 | 12 |
| *ATR R* | 190 | 0.062 | 0.049 | 0.006 | 0.046 | 0.174 | 0.232 | 11 |
| *CG L* | 87 | 0.047 | 0.025 | 0.011 | 0.045 | 0.088 | 0.153 | 11 |
| *CG R* | 87 | 0.056 | 0.033 | 0.013 | 0.043 | 0.113 | 0.167 | 11 |
| *CST L* | 128 | 0.086 | 0.031 | 0.011 | 0.087 | 0.130 | 0.177 | 10 |
| *CST R* | 143 | 0.065 | 0.028 | 0.012 | 0.064 | 0.116 | 0.147 | 8 |
| *FMA L* | 164 | 0.055 | 0.034 | 0.005 | 0.048 | 0.122 | 0.159 | 11 |
| *FMA R* | 39 | 0.057 | 0.027 | 0.022 | 0.053 | 0.109 | 0.147 | 8 |
| *FMI L* | 155 | 0.110 | 0.052 | 0.009 | 0.110 | 0.193 | 0.250 | 13 |
| *FMI R* | 223 | 0.062 | 0.031 | 0.007 | 0.060 | 0.121 | 0.171 | 11 |
| *HPC L* | 37 | 0.072 | 0.028 | 0.027 | 0.072 | 0.115 | 0.140 | 4 |
| *HPC R* | 76 | 0.056 | 0.023 | 0.016 | 0.053 | 0.091 | 0.117 | 11 |
| ***IFF L**** | **137** | **0.114** | **0.052** | **0.002** | **0.111** | **0.208** | **0.222** | **14** |
| *IFF R* | 231 | 0.081 | 0.046 | 0.009 | 0.071 | 0.167 | 0.253 | 10 |
| ***ILF L**** | **208** | **0.084** | **0.050** | **0.009** | **0.074** | **0.183** | **0.231** | **10** |
| *ILF R* | 156 | 0.095 | 0.048 | 0.013 | 0.100 | 0.174 | 0.215 | 10 |
| *SLF-t L* | 10 | 0.071 | 0.030 | 0.034 | 0.067 | 0.115 | 0.138 | 0 |
| *SLF-t R* | 21 | 0.138 | 0.035 | 0.071 | 0.137 | 0.195 | 0.219 | 17 |
| *SLF L* | 217 | 0.093 | 0.048 | 0.006 | 0.090 | 0.166 | 0.250 | 10 |
| *SLF R* | 220 | 0.110 | 0.032 | 0.031 | 0.107 | 0.166 | 0.197 | 17 |
| *UF L* | 144 | 0.085 | 0.050 | 0.007 | 0.087 | 0.165 | 0.208 | 10 |
| *UF R* | 39 | 0.078 | 0.044 | 0.022 | 0.064 | 0.164 | 0.189 | 3 |

Supplementary Table S4 Summary statistics of strain rate across all tracts. Tracts which intercept a microbleed are denoted by *. The post hoc Dunn test with Bonferroni correction is summarised in the final column by sum of tracts with significant difference.

| Tract | n | Mean (*s^-1^)* | Std (*s^-1^)* | Min (*s^-1^)* | 50% (*s^-1^)* | 95% (*s^-1^)* | Max (*s^-1^)* | No. sig dif (Dunn) |
| --- | --- | --- | --- | --- | --- | --- | --- | --- |
| ATR L | 365 | 29.9 | 15.9 | 4.65 | 25.6 | 60.5 | 81.4 | 7 |
| ATR R | 190 | 18.8 | 10.4 | 3.49 | 16.3 | 36.7 | 47.7 | 14 |
| CG L | 87 | 14.6 | 6.08 | 4.65 | 14.0 | 26.5 | 31.4 | 16 |
| CG R | 87 | 16.3 | 9.37 | 5.81 | 14.0 | 31.0 | 66.3 | 16 |
| CST L | 128 | 27.7 | 10.4 | 11.6 | 27.3 | 39.5 | 77.9 | 6 |
| CST R | 143 | 23.7 | 16.3 | 6.98 | 18.6 | 55.8 | 97.7 | 11 |
| FMA L | 164 | 18.2 | 10.4 | 3.49 | 16.3 | 38.2 | 53.5 | 14 |
| FMA R | 39 | 21.2 | 9.41 | 6.98 | 19.8 | 35.5 | 50.0 | 5 |
| FMI L | 155 | 39.4 | 18.2 | 4.65 | 39.5 | 68.6 | 93.0 | 14 |
| FMI R | 223 | 27.4 | 14.4 | 3.49 | 25.6 | 58.8 | 83.7 | 6 |
| HPC L | 37 | 25.1 | 10.4 | 10.5 | 23.3 | 46.5 | 50.0 | 3 |
| HPC R | 76 | 36.0 | 18.6 | 10.5 | 32.6 | 69.5 | 114 | 8 |
| **IFF L*** | **137** | **37.0** | **17.1** | **4.65** | **34.9** | **66.3** | **81.4** | **11** |
| IFF R | 231 | 25.3 | 10.8 | 5.81 | 25.6 | 45.3 | 52.3 | 8 |
| **ILF L*** | **208** | **28.8** | **18.3** | **3.49** | **24.4** | **62.4** | **96.5** | **7** |
| ILF R | 156 | 34.0 | 20.7 | 6.98 | 29.1 | 69.2 | 122 | 6 |
| SLF-t L | 10 | 20.8 | 9.61 | 11.6 | 18.0 | 37.3 | 43.0 | 0 |
| SLF-t R | 21 | 31.1 | 8.39 | 16.3 | 30.2 | 43.0 | 53.5 | 4 |
| SLF L | 217 | 27.8 | 13.4 | 2.33 | 27.9 | 47.7 | 87.2 | 7 |
| SLF R | 220 | 30.0 | 10.4 | 10.5 | 27.9 | 50.0 | 61.6 | 7 |
| UF L | 144 | 31.6 | 18.8 | 4.65 | 26.7 | 65.1 | 83.7 | 6 |
| UF R | 39 | 31.7 | 19.9 | 9.30 | 29.1 | 65.0 | 96.5 | 4 |
